# Outbreak of Sporotrichosis, Western Australia

**DOI:** 10.3201/eid1308.061462

**Published:** 2007-08

**Authors:** Kynan T. Feeney, Ian H. Arthur, Amanda J. Whittle, Shelley A. Altman, David J. Speers

**Affiliations:** * Sir Charles Gairdner Hospital, Nedlands, Western Australia, Australia; † PathWest Laboratory Medicine, Nedlands, Western Australia, Australia; ‡ South West Population Health Unit, Bunbury, Western Australia, Australia

**Keywords:** Sporothrix schenckii, sporotrichosis, hay, Western Australia, dispatch

## Abstract

A cluster of sporotrichosis cases occurred in the Busselton-Margaret River region of Western Australia from 2000 to 2003. Epidemiologic investigation and mycologic culture for *Sporothrix schenckii* implicated hay initially distributed through a commercial hay supplier as the source of the outbreak. Declining infection rates have occurred after various community measures were instigated.

Sporotrichosis is an infection caused by the fungal species *Sporothrix schenckii*. It predominantly causes subacute to chronic subcutaneous infection, which occurs when the fungus enters small breaks in the skin (*1*). *S. schenckii* is widely found on organic material in the environment such as sphagnum moss, fruits, and plants. Human cases have been reported from intermediate hosts; cats were the source of an outbreak in Rio de Janeiro (*1*–*3*). Outbreaks in Australia and other countries have been previously linked to contact with hay (*4*,*5*).

In Western Australia, sporadic cases have occurred for many years in the southwest, particularly in the wheat-growing areas (Figure 1), but in the year 2000 an increase in the number of cases of sporotrichosis was noted (*6*). Forty-one microbiologically confirmed human cases were reported from 2000 to 2003 compared with 8 cases from 1997 to 1999 at the PathWest Laboratory at QEII Medical Centre, which has branches throughout metropolitan and regional Western Australia. A review of these cases found that 22 cases were from the Busselton-Margaret River (BMR) region of Western Australia, where no cases had previously been recorded.

**Figure 1:**
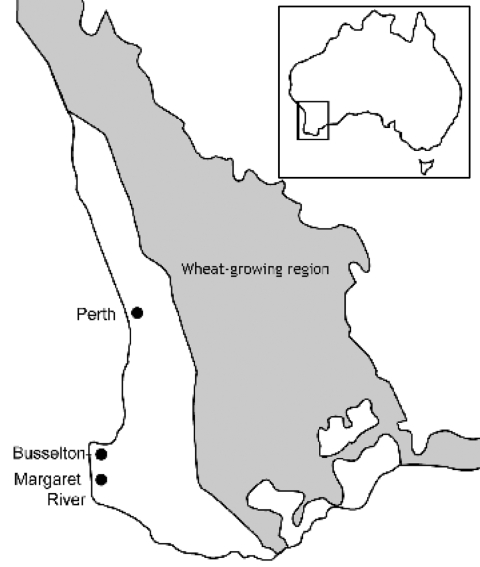
Wheat-growing region and Busselton-Margaret River region of Western Australia.

## The Study

An epidemiologic study was begun in 2003 to determine the nature and source of the infection. Pathology laboratories and general practitioners in the southwest region of Western Australia were contacted to ascertain patients with cases of sporotrichosis, defined as clinical evidence of disease supported by microbiologic confirmation. Telephone interviews, which included questions on possible sources of infection, were conducted with all identified patients.

The epidemiologic study (Table 1) discovered 11 patients with a microbiologic diagnosis of sporotrichosis from July 2003 to July 2004 in the southwest region. All lived in the BMR region except for 2 (patients 3 and 5).

**Table 1:** Sporotrichosis cases in Western Australia, July 2003–July 2004

Patient no.	Sex	Age, y	Area of residence	Pre-existing cuts	Onset of symptoms, mo/y	Duration of disease, mo	Antifungal treatment	Suspected exposure*	GenBank accession no.
1	F	57	BMR†	Present	7/03	6	Surgery, oral traconazole, oral K‡	Camping trip in a different region of Australia	EF589115
2	M	48	BMR	Present	10/03	5	Oral traconazole	Hay (2)	EF589117
3	M	68	Greenbushes	Present	11/03	4	Oral traconazole	Gardening	EF589116
4	F	39	BMR	Present	12/03	4	Oral traconazole	Hay (1)	EF589119
5	M	65	Collie	Present	12/03	4	Oral traconazole	Hay (own supply)	Not available
6	M	56	BMR	Absent	12/03	4	Oral traconazole	Hay (1)	EF589121
7	F	69	BMR	Absent	1/04	2	Oral traconazole	Hay (1)	EF589118
8	M	66	BMR	Absent	1/04	3.5	Oral traconazole	Hay (2)	EF589120
9	M	10	BMR	Present	2/04	2	Surgery, oral traconazole	Hay (1)	EF589122
10	F	8	BMR	Present	7/04	5	Oral traconazole	Hay (1)	EF589124
11	F	3	BMR	Present	7/04	5	Naturopathic treatment	Hay (1)	EF589123

*1 and 2 refer to outlet 1 and 2, respectively. †Busselton-Margaret River region. ‡Saturated solution of potassium iodide.

Nine of the 11 case-patients had contact with hay preceding the development of sporotrichosis; 8 had bought the hay from 1 of 2 commercial suppliers located in the township of Margaret River (outlet 1 and outlet 2). The remaining patient had contact with hay grown on his property. Seven of the 9 persons with cases linked to hay exposure had used the hay for domestic gardening; the other 2 patients were exposed to hay used for commercial farming. Patients 10 and 11 were children who played together in hay purchased from outlet 1 before the onset of symptoms of sporotrichosis. Patients 1 and 3 had no documented hay exposure; sporotrichosis developed after a camping trip and after gardening, respectively.

Nine case-patients were initially treated with oral antimicrobial agents for a presumed bacterial infection. Because of a lack of clinical response, wound swabs or biopsy specimens were obtained. Sporotrichosis was subsequently diagnosed, and antifungal therapy was then begun. Four patients required inpatient treatment for 1 of several reasons: initially to receive intravenous antimicrobial agents before the diagnosis, to undergo debridement surgery and biopsy, or to treat complications related to therapy. Ten of the 11 patients received oral itraconazole; 1 of these patients also received a saturated solution of potassium iodide, and 1 patient ceased oral antifungal therapy because of severe side effects. One of the 11 received only a naturopathic remedy.

When hay was implicated as a likely source, local environmental health officers visited commercial hay outlets in the area to assess procurement, storage, and distribution practices. Fifty hay samples were collected from around the BMR region for mycologic culture. Sixteen samples were from properties associated with 6 of the cases, 9 from the 2 commercial hay outlets, 7 from a farm that supplied hay to a commercial outlet, and 18 from other properties in the BMR region not associated with the outbreak (control samples).

Mycologic culture for organisms from the *Ophiostoma–Sporothrix* complex was performed on a portion of each hay sample. Isolates were examined for morphologic features and analyzed by pulsed-field gel electrophoresis (PFGE) (*7*). Sequencing the ITS1 region with universal primers *ITS1* and *ITS2* and the ITS2 region with universal primers *ITS3* and *ITS4* was performed with Applied Biosystems BigDye Terminator v3.1 Cycle Sequencing reagent using the Applied Biosystems Prism 3100 Avant Genetic Analyzer (Foster City, CA, USA). Using ITS sequencing, de Beer et al. (*8*) distinguished clinical strains of *S. schenckii* from environmental strains.

Isolates positive for the clinical group of *S. schenckii* showed morphologic features of conidia that are predominantly oval and display sleeves of dematiaceous conidia along the hyphae. When BLAST software (www.ncbi.nlm.nih.gov/blast) was used, these isolates had ITS2 sequences that conform to the clinical group noted by de Beer et al. (*8*). They differ from Western Australian environmental isolates of the *Opiostoma–Sporothrix* complex obtained in this study and clinical *S. schenckii* isolates from the eastern states of Australia in their morphologic features, PFGE patterns (*7*), and ITS2 sequences (data not shown).

Of the 6 case-patient–related properties from which environmental samples were taken (Table 2), 3 had a single sample that was culture positive for *S. schenckii* (clinical strain). Each of these 3 patients had purchased hay from the same commercial hay outlet (outlet 1). One of the hay samples from outlet 1 was also culturally positive for *S. schenckii* clinical group. These 4 isolates had identical ITS2 sequences and morphologic features that were similar to all our patient isolates available for testing (the patient 5 isolate was not available). *S. schenckii* (clinical strain) was not isolated from any of the control hay samples, although other members of the *Opiostoma–Sporothrix* complex were isolated in many environmental samples.

**Table 2:** Culture findings from environmental sampling

Hay sample source	No. samples	Samples culture positive for Sporothrix schenckii* (GenBank accession no.)
Hay used by case-patient 2	2	0
Hay used by case-patient 4	2	1 (EF589128)
Hay used by case-patient 6	3	1 (EF589126)
Hay used by case-patient 7	4	0
Hay used by case-patient 8	3	1 (EF589125)
Hay used by case-patient 9	2	0
Hay outlet 1	5	1 (EF589127)
Hay outlet 2	4	0
Source of outlet 1	7	0
Control samples	18	0

*S. schenckii clinical group as described in text.

## Conclusions

The epidemiologic aspect of our study implicated contaminated hay as the source of an outbreak of sporotrichosis in the BMR region. Exposure was documented for 9 of the 11 case-patients. Most patients described contact with hay during gardening. Eight patients had contact with hay purchased at commercial suppliers in the Margaret River region.

Mycologic culture of hay samples confirmed that the hay was a possible source of infection. Half of the case-related properties tested were culture positive for the clinical strain of *S. schenckii,* as was a sample from a commercial hay supplier that had supplied hay to these properties. None of the control samples or samples from a farm that supplied hay to this hay supplier showed *S. schenckii* (clinical strain) on mycologic culture.

All Western Australia clinical isolates tested—including the BMR outbreak isolates, the isolates from the hay supplier, and 3 case-related hay samples—are indistinguishable by ITS2 sequencing and PFGE. These isolates differ from the environmental isolates of the *Ophiostoma*–*Sporothrix* complex tested from our survey and from eastern states’ clinical isolates. Therefore, the epidemiologically implicated hay from the commercial hay suppliers was considered the likely source of the regional outbreak.

**Figure 2:**
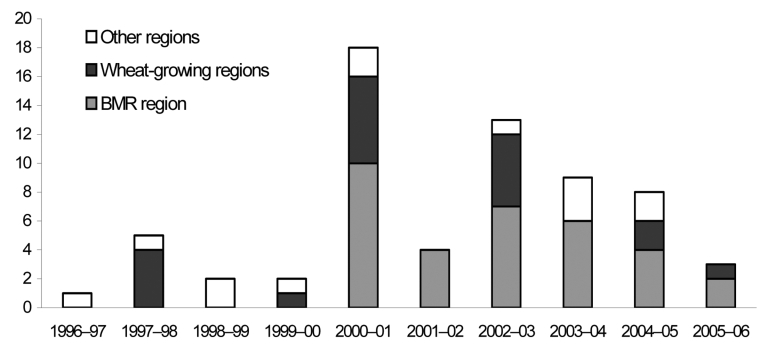
Sporotrichosis clinical isolation data from PathWest (QEII), July 1996–June 2006. BMR, Busselton-Margaret River.

This finding prompted intervention in the community. Commercial hay suppliers cooperated by destroying any moldy hay on their properties and storing hay awaiting sale on rubber matting under cover. Information about the diagnosis and management of the infection was distributed to general practitioners in the area, and general information was distributed to the community through various sources such as community newspapers. Since the initial outbreak of sporotrichosis in the BMR region, the infection rate has decreased (Figure 2). Further distribution of the organism by hay suppliers across the region appears to have ceased with the introduction of infection control measures.
